# Does receiving high or low breast cancer risk estimates produce a reduction in subsequent breast cancer screening attendance? Cohort study

**DOI:** 10.1016/j.breast.2022.05.001

**Published:** 2022-05-09

**Authors:** David P. French, Lorna McWilliams, Anthony Howell, D Gareth Evans

**Affiliations:** a Manchester Centre of Health Psychology, Division of Psychology and Mental Health, School of Health Sciences, University of Manchester, Coupland Street, Manchester, M13 9PL, England, UK; b NIHR Manchester Biomedical Research Centre, Manchester Academic Health Science Centre, Central Manchester University Hospitals NHS Foundation Trust, Manchester, England, UK; c The Nightingale and Prevent Breast Cancer Centre, Manchester University NHS Foundation Trust, Manchester, M23 9LT, England, UK; d Manchester Breast Centre, Manchester Cancer Research Centre, University of Manchester, 555 Wilmslow Rd, Manchester, M20 4GJ, England, UK; e Department of Medical Oncology, The Christie NHS Foundation Trust, Wilmslow Rd, Manchester, M20 4BX, England, UK; f Genomic Medicine, Division of Evolution and Genomic Sciences, The University of Manchester, St Mary's Hospital, Manchester University NHS Foundation Trust, Manchester, M13 9WL, England, UK

**Keywords:** Risk stratification, Screening, Attendance, Risk communication, Breast cancer, Risk adaptation, Screening harms

## Abstract

Risk-stratified breast cancer screening may improve the balance of screening benefits to harms.

We assess a potential new harm: reduced screening attendance in women receiving below average-risk (false reassurance) or higher-risk results (screening avoidance). Following initial screening, 26,668 women in the PROCAS study received breast cancer risk estimates, with attendance recorded for two subsequent screening rounds. First-screen attendance was slightly reduced in below-average (85.6%) but not higher-risk women, compared to other women (86.4%). Second-screen attendance increased for women at higher-risk (89.2%) but not below-average, compared to other women (78.8%). Concerns about this potential harm of risk-stratified screening therefore appear unfounded.

## Highlights

• A potential harm of risk-stratified screening is a reduction in future attendance.
• Some evidence of a small reduction in future attendance in below-average women.
• Large increase in attendance in higher-risk women at future screening rounds.

## Abbreviations

NHS-BSP: National Health Service Breast Screening Programme
PROCAS: (Predicting Risk Of Cancer At Screening Study) prospective cohort study

## 1 Introduction

Breast cancer screening, in common with all screening, involves harms such as false positive screening test results and overdiagnosis, as well as benefits, notably, reducing breast cancer mortality [1]. One possible means of improving the balance of benefits to harms is to stratify screening according to individual cancer risk, where women at high-risk may be offered more frequent screening and preventive therapies [2] (see Table 1).

However, risk stratified screening could introduce new potential harms [3]. Telling some women that they are at below-average risk may produce false reassurance, whereby screening is no longer deemed necessary. Conversely, telling some women that they are at high-risk may produce avoidance whereby those women no longer attend screening as it is too anxiety-provoking. We have previously reported no effects of receiving risk estimates on uptake of subsequent screening appointments relative to rates of screening attendance in those sites outside of the study period [4]. However, internationally no data have yet been reported on whether receiving risk estimates produces adverse effects on subsequent cancer screening attendance, either in lower-risk (“false reassurance”) or higher-risk (“avoidance”) women.

The present study examines this potential harm with women who were provided with risk estimates in the PROCAS (Predicting Risk Of Cancer At Screening Study) prospective cohort study [5].

## 2 Methods

### 2.1 Design

A prospective cohort study with women who attended the NHS-BSP and received breast cancer risk estimates due to participation in PROCAS. Attendance at the two subsequent rounds of the Greater Manchester NHS Breast Screening Programme (NHS-BSP) was predicted by risk category estimated.

### 2.2 Participants and procedure

Women were aged 46–73 years at initial consent to participate in the PROCAS study (09/2009–08/2014). Of the 127,000 women invited into the PROCAS Study, 70,000 attended screening, and 58,000 accepted the offer of risk estimation [5]. Estimates were produced by the Tyrer-Cuzick model [6], which incorporated self-reported information about family history, lifestyle and hormonal factors, as well as breast density from mammograms, and, in a sub-sample, single nucleotide polymorphisms identified via saliva samples. Women received the following 10-year risk estimates: high (>8%), moderate (5–7.99%), average (2–4.99%) or below-average (<2%).

The analysis included, a random selection of 11,300 women told they were at average/below-average and all women told they were at moderate/high risk. Women who died, did not receive risk feedback, or made NHS-BSP appointments before risk estimates were received were all excluded. Risk feedback was provided 2–4 years (2011–2016) after initial consent and provision of information to estimate breast cancer risk. For the second screen following risk assessment, only women who received risk feedback before 2015 were included. Repeat NHS-BSP attendance took place from (07/2012–03/2020), with the last mammogram before April 01, 2020. The present analysis was based on NHS-BSP records of appointments that were booked and attended, as well as attendance at enhanced screening appointments for high-risk women at the Nightingale Breast Screening Centre.

### 2.3 Analysis

Logistic regression was used to examine the extent to which rates of attendance at (i) first screening appointment and (ii) second screening appointment following provision of risk information, were predicted by breast cancer risk communicated.

## 3 Results

Of the 26,680 women eligible for the present study, 23,052 (86.4%) attended their first NHS-BSP appointment following the provision of risk information. Of the 13,139 women eligible for a second mammogram after receiving risk estimation, 10,342 (78.8%) attended.

Women at below average risk were slightly less likely to attend for their first screening appointment (85.6%) than other groups (β = −0.115,SE(β) = 0.036, Wald χ^2^ = 10.20,df = 1,p = 0.001,OR = 0.89). There was no evidence that women at below average risk were less likely to attend for their second screening appointment (78.5%) than other groups (β = −0.035,SE(β) = 0.043, Wald χ^2^ = 0.665,df = 1,p = 0.415,OR = 0.97) (Table 1).

**Table 1:** Rates of attendance at first and second breast cancer screening appointments, according to previously estimated breast cancer risk.

	Number of women eligible (first screen)	Number attended first screen (percentage of women eligible)	Number of women eligible (second screen)	Number attended second screen (percentage of women eligible)
Below average	11,293	9669 (85.6%)	5528	4339 (78.5%)
Average	11,293	9844 (87.2%)	5569	4346 (78.0%)
Moderate	3368	2916 (86.6%)	1652	1327 (80.3%)
High	714	623 (87.3%)	370	330 (89.2%)
Total	26,668	23,052 (86.4%)	13,119	10,342 (78.8%)

Women at high-risk were not significantly less likely to attend for either their first screening appointment (87.3%) than other groups (β = 0.077,SE(β) = 0.114, Wald χ^2^ = 0.454,df = 1,p = 0.50,OR = 1.08). Moreover, they were more likely to attend (89.2%) their second screening appointment (β = 0.813,SE(β) = 0.169, Wald χ^2^ = 23.21,df = 1,p < 0.001,OR = 2.26).

This pattern of findings was unaffected by adjusting for age at screening and duration between original screening appointment and being sent risk estimates.

## 4 Discussion

The present results indicate a slightly lower attendance at the next screen in women who received below average risk estimates, but no significant difference at the second screen. For women who received an estimate of high breast cancer risk, uptake was significantly increased at the second screen.

The present sample of women are a somewhat atypical group in terms of attendance, as they all initially attended the NHS-BSP to be eligible for the PROCAS study (70% of eligible women attended screening), and also as they consented to take part in the PROCAS study (38% of attendees) [4]. They are therefore likely to be more in favour of screening than the general population, who would be expected to be more ambivalent about screening. This attitudinal variable was not assessed nor were other variables often found to be linked to screening attendance such as ethnicity, due to the use of routinely collected data by the NHS-BSP. Despite this, the present sample were appropriate to address the present research question regarding screening re-attendance. Further, attendance rates were similar to a directly comparable sample of women from the Greater Manchester NHS-BSP who attended their previous mammogram and whose last screen was within the previous five years: in 2012–2013 39,058 women were invited and 32,925 (84.3%) attended [5].

The present study found “false reassurance” effects of very small magnitude, with 85.6% of women at below-average risk attending their first screen, compared to an overall sample rate of 86.4%. Similarly, there is no evidence of “avoidance”, with higher-risk women showing a much greater likelihood to attend the second screen offered (89.2% attendance). These findings of little impact on behaviour are in line with previous research [7] including a questionnaire study with PROCAS participants, which found no evidence of changing other health-related behaviours according to risk estimate received [8].

There is no reason to expect a major reduction in NHS-BSP attendance for groups of women who receive either below-average or high-risk estimates. This adds to the body of evidence suggesting that many concerns about harms of risk stratified screening, such as receiving risk estimates producing anxiety are often unfounded [9].

## Ethics approval and consent to participate

The present research was approved by Liverpool East NHS Research Ethics Committee [14/NW/1445]. All participants gave written informed consent for their data to be used in the present publication as part of the PROCAS study. The study was performed in accordance with the Declaration of Helsinki.

## Consent for publication

All participants gave written informed consent for their data to be used.

## Availability of data and materials

The datasets generated and/or analysed during the current study are not publicly available due to including information that could potentially allow individuals to be identified, but are available from the corresponding author on reasonable request.

## Funding

This article presents independent research funded by the 10.13039/501100000272National Institute for Health Research (10.13039/501100000272NIHR) Programme Grants for Applied Research programme, reference numbers RP-PG-0707-10,031: “Improvement in risk prediction, early detection and prevention of breast cancer” and Ref: RP-PG-1214-20,016: “Providing breast cancer risk information as part of national breast cancer screening programme: building an evidence base on benefits and harms to inform a decision to implement (PROCAS2).” All authors are supported by the 10.13039/501100000272NIHR 10.13039/100014461Biomedical Research Centre in Manchester (IS-BRC-1215-20,007). The views expressed are those of the author(s) and not necessarily those of the 10.13039/501100000272NIHR, or the 10.13039/501100000276Department of Health and Social Care.

## Authors’ contributions

**David P French** conceived this add-on to the main PROCAS study, and all authors contributed to the study design. Funding was acquired by **D Gareth Evans** as part of larger programmes of work. **D Gareth Evans** organised the study and oversaw data extraction from the relevant datasets. **David P French** conducted all analyses. **David P French** drafted the manuscript, and all authors contributed towards interpretation of data and writing and review of the manuscript. All authors have read and approved the final version of the manuscript. The corresponding author attests that all listed authors meet authorship criteria and that no others meeting the criteria have been omitted.

## Declaration of competing interest

The authors declare they have no competing interests.
